# The Effects of Dietary *Saccharomyces cerevisiae* Supplementation on Gut Microbiota Composition and Gut Health in Aged Labrador Retrievers

**DOI:** 10.3390/ani14121713

**Published:** 2024-06-07

**Authors:** Yingyue Cui, Deping Li, Mingrui Zhang, Pan Liu, Haotian Wang, Yingying Li, Yi Wu

**Affiliations:** 1 State Key Laboratory of Animal Nutrition and Feeding, College of Animal Science and Technology, China Agricultural University, Beijing 100193, China; sy20233040846@cau.edu.cn (Y.C.); zhangmingrui@cau.edu.cn (M.Z.); liup@cau.edu.cn (P.L.); wanghaotian@cau.edu.cn (H.W.); lyying@cau.edu.cn (Y.L.); 2 Hangzhou Netease Yanxuan Trading Co., Ltd., Hangzhou 310051, China; lideping@corp.netease.com

**Keywords:** *Saccharomyces cerevisiae*, gut health, gut microbiota, immunity, antioxidant capacity, aged dogs

## Abstract

**Simple Summary:**

*Saccharomyces cerevisiae* is a functional probiotic known to positively impact intestinal health in humans and other mammals. However, its effects on gut health in aged dogs have not been thoroughly studied. In our study, we investigate the effects of dietary *Saccharomyces cerevisiae* supplementation on the gut barrier, immune functions, antioxidant capacity, and gut microbiota profile in aged dogs. The results suggest that the addition of active *Saccharomyces cerevisiae* may improve the gut microbiota structure, enhance immune function, and increase the antioxidant capacity in aged dogs.

**Abstract:**

The intestinal microbiome changes with age, influencing the host’s health and immune status. *Saccharomyces cerevisiae* (*S. cerevisiae*) positively affects intestinal function in humans and animals, but its effects on gut health and the microbiota profile in aged dogs have not been thoroughly investigated. Twenty aged Labrador Retrievers were divided into two groups: a control group (CON) and a *S. cerevisiae* group (SC). The experiment lasted for 42 days, with assessments of their intestinal barrier function, inflammatory factors, antioxidant markers, and fecal microbiome composition. The results showed that dietary *S. cerevisiae* reduced the levels of TNF-α, IL-6, and IL-1β in the serum (*p* < 0.05). In the SC group, plasma superoxide dismutase and glutathione peroxidase activities increased, while the level of malondialdehyde significantly decreased (*p* < 0.05). Additionally, dietary *S. cerevisiae* lowered the serum zonulin and lipopolysaccharide (LPS) levels (*p* < 0.05) and inhibited fecal ammonia production (*p* < 0.05). Furthermore, the microbiota profile showed that dietary *S. cerevisiae* decreased the abundance of Firmicutes but increased the Chao index, the abundance of Bacteroidetes, and the proportion of Bacteroidetes to Firmicutes (*p* < 0.05). To conclude, dietary *S. cerevisiae* can regulate the gut’s microbial structure and gut health, which may contribute to the overall health of companion animals as they age.

## 1. Introduction

The gastrointestinal tract houses complex microbiota that impact the health of humans and animals [1]. The gut microbiota help maintain host health through multiple mechanisms, such as improving nutrient absorption, defending against intestinal pathogens, enhancing barrier function, and suppressing inflammatory progression [2]. In healthy dogs, the core flora of the intestinal microbiota at the phylum level are dominated by Actinobacteria, Fusobacteria, Bacteroidetes, Proteobacteria, and Firmicutes [3]. Abnormal alterations in the intestinal flora in dogs have been found to be related to intestinal-related diseases, including acute hemorrhagic diarrhea syndrome, and chronic enteropathy, such as inflammatory bowel disease. Changes in the microbial metabolite profile and a decrease in microbial diversity are markers of microbial dysbiosis [4,5]. Notably, such alterations lead to damage in the gut barrier and subsequently induce gut inflammation, which is characterized by an increase in pro-inflammatory cytokines, including interleukin (IL)-1β and tumor necrosis factor-α (TNF-α) [4,5].

From birth, the gut microbiota co-evolve with animal age and drive the maturation of the intestinal immune state. Furthermore, age-related alterations in the microbial communities are related to intestinal immune and host metabolism disorders [6]. Puppies have immature microbiota in the first few weeks of life, but by about 4–5 months of age, the gut flora become similar to those in adulthood and remain relatively stable until old age [7]. Aged dogs exhibit an increase in the abundance of Ruminococcaceae, Bacteroidaceae, Veillonellaceae, and Lachnospiraceae, accompanied by reduced production of valerate and butyrate [8]. In addition, the increased abundance of Bacteroidetes and its order Bacteroides, as well as the decreased abundance of Firmicutes and its genus Streptococcus, are associated with improved intestinal immunity in aged dogs [9]. Therefore, the gut microbiota is essential to the overall health of aged dogs, and effective strategies based on gut microbes should be explored to improve the health of dogs during the aging process.

Probiotics are an effective method for manipulating the intestinal flora to support the health of the host and have been explored as nutritional supplements for humans and farm animals [10,11]. Probiotics benefit the physiological condition of the host by interacting with the gut microbiota and communicating with the intestinal epithelial cells, which can maintain immune balance by enhancing the integrity of the epithelial barrier [12]. Furthermore, probiotics have been shown to improve feces quality and reduce the fecal concentration of certain nitrogen fermentative catabolites and dogs’ fecal odor [13,14]. Additionally, a multi-strain probiotic compound has also been suggested to improve nutrient digestibility, leading to an increase in feed intake and body weight in dogs [15,16]. In dogs with acute hemorrhagic diarrhea syndrome, studies have found that an oral probiotic compound promoted the normalization of the gut microbiota and clinical recovery. Additionally, in dogs with idiopathic inflammatory bowel disease, probiotic supplements have been shown to elevate the levels of intestinal tight junction proteins [17,18].

Probiotics based on *Saccharomyces cerevisiae* have been found to be functional dietary additives that positively affect gut function for humans and animals by reducing inflammation and regulating the gut microbiota [19,20]. Furthermore, as an essential component of the cell wall of *S. cerevisiae*, functional polysaccharides, including mannooligosaccharides (MOS) and β-glucan, can trigger the immune response by specifically binding to immune cell receptors or by directly altering the composition of intestinal microorganisms and their metabolites [21]. It has been reported that consuming *S. cerevisiae* for 35 days can significantly improve fecal consistency in adult dogs [22]. Moreover, feeding dogs with *S. cerevisiae* yeast for 49 days not only resulted in reductions in the gut microbiota dysbiosis index, total biogenic amine levels, and ammonia concentration but also increased the abundance of *Turicibacter* and *Bifidobacteria* and decreased the abundance of *Escherichia coli* and *Lactobacillus* [14]. However, previous studies have not investigated the impact of *S. cerevisiae* intake on aged dogs.

Therefore, this study aimed to investigate the influence of dietary supplementation of *S. cerevisiae* on the gut barrier, antioxidant capacity, immune response, and gut microbiota profile in aged dogs.

## 2. Materials and Methods

### 2.1. Animals and Feeding Management

The Institutional Animal Care and Use Committee of China Agricultural University approved the experimental protocols for the dietary treatments and animal handling (Experiment identification code: AW50503202-2-2).

Before commencing the study, all the Labrador Retrievers were medically examined to determine their eligibility for the trials. The examinations included parasite analysis, body weight (BW), body condition score (BCS), and fecal score. The parasites examined included fleas, ticks, lice, hair follicle worms, ear mites, scabies mites, heartworms, coccidia, toxoplasma, roundworms, hookworms, whipworms, tapeworms, and nematodes. The BCS was assessed using a nine-point scale [23]. Fecal samples were evaluated using a five-point scale as follows: 1 = hard, dry pellets; small hard mass; 2 = hard, formed, dry stool; remains firm and soft; 3 = soft, formed, and moist stool; retains shape; 4 = soft, unformed stool; assumes shape of container; 5 = watery; liquid that can be poured. There were no abnormalities in the above indicators, and no parasitic infections were found in any of the dogs. The animals did not receive any drugs or diets related to the test function, and no surgical procedures, immunosuppressive drugs, or antibiotics were administered during the three months prior to study enrollment. Dogs that could not take oral feed, had chronic medical conditions, or were nursing or pregnant were excluded. During the experiment, each Labrador Retriever was housed individually in a 1.2 × 1.5 m^2^ room. Table 1 presents the diet’s ingredients and nutritional concentrations. The basal diet met the nutritional requirements recommended by the NRC (2006) for adult dogs.

### 2.2. Experimental Design and Sample Collection

All 20 Labrador Retrievers, with a mean age of 9.32 ± 1.71 years and a mean BW of 28.74 ± 0.87 kg, were divided into two groups with 10 animals per treatment, consisting of 5 females and 5 males. The experiment lasted for 42 days. During the experiment period, the dietary groups were as follows: (1) Control group: Dogs were fed the basal diet without *S. cerevisiae* supplementation (CON); (2) *S. cerevisiae* group: Dogs were fed the same diet supplemented with 2 × 10^10^ CFU/kg *S. cerevisiae* (SC).

On days 21 and 42, their feed intake and BW were cataloged. On days 0, 21, and 42, 5 mL of blood was extracted from the small saphenous vein of the lateral hind limb of each dog, and the serum was separated from 3 mL of blood using centrifugation for 20 min at 3000 rpm and 4 °C and then stored at −20 °C for ELISA analysis. On days 0, 21, and 42, part of a fresh fecal sample was collected immediately for each dog and then stored at −20 °C for analyses of branched chain fatty acids (BCFAs), short chain fatty acids (SCFAs), and ammonia. Another part of the fresh fecal sample collected immediately was stored at −80 °C for bacterial analyses. On days 22 (8:00 am) to 27 (8:00 am), the total feces were collected from each dog and kept at −20 °C until detection of the apparent total tract digestibility (ATTD, %) of the nutrients.

### 2.3. Total Tract Apparent Digestibility of Nutrients

A total of 5 g of the fecal samples and feed was determined using the methods of the Association of Official Analytical Chemists for ether extract (EE; method 920.39), ash (method 942.05), crude protein (CP; method 984.13), and dry matter (DM; method 930.15). Gross energy (GE) was measured using an automatic adiabatic oxygen bomb calorimeter (Parr 6400, Calorimeter, Moline, IL, USA). Organic matter (OM) was determined as the difference between ash and DM. The ATTD was assessed using the following equation:

ATTD (%) = [nutrient intake (g/d) − fecal output (g/d)] × 100/nutrient intake (g/d)

### 2.4. Fecal Microbiota Analysis

DNA extraction from the dog feces involved the utilization of the QIAamp Fast DNA Stool Mini Kit (QIAGEN, Hilden, Germany). The amplification of the V3-V4 region of the 16S rRNA employed the universal primers 338F (5′-ACTCCTACGGGAGGCAGCAG-3′) and 806R (5′-GGACTACHVGGGTWTCTAAT-3′), followed by equimolar pooling and sequencing on the Illumina MiSeq platform to generate 300-base-pair (bp) paired-end reads. Quality control of the raw 16S rRNA sequences was conducted using fastp software (version 0.19.6), while splicing was performed using FLASH software (version 1.2.11). Subsequently, UPARSE software (Version 11) was utilized to cluster the optimized sequences into operational taxonomic units (OTUs) at a 97% sequence similarity level. Taxonomic classification of each OTU representative sequence was achieved using the RDP Classifier (Version 2.2) against the Silva138 database with a confidence threshold of 0.7. Alpha diversity indices, including the Chao index, the Simpson index, and the Shannon index, were computed using mothur (Version 1.30.2). Beta diversity was assessed according to Bray-Curtis distance and illustrated via principal coordinate analysis (PCoA). The taxonomic composition of community species in each sample was analyzed across all taxonomic levels.

### 2.5. Fecal Metabolite Concentrations

The fecal concentrations of SCFAs and BCFAs were assessed via gas chromatography–mass spectrometry (GC-MS) at Shanghai Major Biomedical Technology Co., Ltd., Shanghai, China. Initially, a standard solution containing SCFAs and BCFAs was prepared. Subsequently, 100 mg of the fresh fecal samples was weighed and combined with 450 μL of methanol, along with 50 μL of 2-ethylbutyric acid (at a concentration of 1000 μg/mL), for cryogenic sonication (Ningbo Scientz Biotechnology Co., Ltd., Ningbo, China). Following this, centrifugation was performed at 13,000× *g* for 15 min to isolate the supernatant (Shanghai Eppendorf Biotechnology International Trade Co., Ltd., Shanghai, China). The supernatant was then treated with 50 mg of anhydrous sodium sulfate and centrifuged at 4 °C for 15 min at 13,000× *g* post-edging. The resulting supernatant solution was collected and subjected to analysis conform to the 8890B-5977B GC/MSD system (Agilent Technologies Inc., Santa Clara, CA, USA). The fecal ammonia levels were determined using the method of Chaney and Marbach [24]. Briefly, suspend 2 g of the feces sample in 10 mL of distilled water and create a homogeneous mixture. Centrifuge the mixture and collect the supernatant for analysis. Combine 1 mL of the supernatant in a clean test tube with 1 mL of 0.5% phenol solution, 1 mL of 0.5% sodium hypochlorite solution, and 1 mL of buffer solution to stabilize the pH at approximately 7.4. Keep the treated samples at room temperature for 10 min, and measure the absorbance of the resulting mixture at 640 nm using a spectrophotometer (BioTek Instruments, Inc., Beijing Representative Office, Beijing, China). The concentration of ammonia was assessed using the following equation:

Ammonia (μmol/g) = total ammonia (μmol)/weight of fecal sample (g)

### 2.6. Antioxidant Parameters and Inflammation Indicators

The serum catalase (CAT), malondialdehyde (MDA), superoxide dismutase (SOD), total antioxidant capacity (T-AOC), and glutathione peroxidase (GSH-Px) levels were determined to assess the antioxidant capacity. Additionally, the inflammatory factors in the serum included IL-10, IL-6, IL-1β, and TNF-α. According to the manufacturer’s instructions (Shanghai Enzyme-linked Biotechnology Co., Ltd., Shanghai, China), ELISA kits were applied to determine all the antioxidant parameters and inflammation indicators. The spectrophotometer (BioTek Instruments, Inc., Beijing Representative Office, Beijing, China) was used for reading the data.

### 2.7. Gut Barrier Function Parameters

ELISA kits were utilized to measure the concentrations of diamine oxidase (DAO), intestinal fatty acid-binding protein (i-FABP), zonulin, and lipopolysaccharide (LPS), according to the manufacturer’s instructions. All the kits used in this study were obtained from Shanghai Enzyme-linked Biotechnology Co., Ltd., located in Shanghai, China, and the data read using the spectrophotometer (BioTek Instruments, Inc., Beijing Representative Office, Beijing, China).

### 2.8. Statistical Analysis

Analysis of the data was conducted using IBM SPSS Statistics 26 (Chicago, IL, USA) and GraphPad Prism (version 8.3.0, San Diego, CA, USA), using the R tool to visualize the microbial sequencing data. Bar graphs illustrating the bacterial community were generated by employing the R ggplot package (version 3.3.1), while heatmaps were generated by utilizing the R vegan package. The hematology indexes and comparison of the intestinal microbiome abundance were executed using the Kruskal-Wallis H test.

The data analysis involved employing the Mixed Models procedure in SAS (version 9.4; SAS Institute, Cary, NC, USA). Fixed effects included the time, diet, and their interaction, with each individual dog randomly allocated. The mean of the groups was calculated using the LSMEANS statement, and statistical differences among treatments were detected using the PDIFF option. The digestibility data underwent comparison via one-way ANOVA with Tukey’s post hoc test. A significance threshold of *p* < 0.05 was utilized to ascertain the statistical significance.

## 3. Results

### 3.1. Body Weight and Body Condition Score

The BW, average daily feed intake, and BCSs of the aged dogs are shown in Table 2. The average daily feed intake of the aged dogs was not affected between treatments or during the experimental period (*p* ≥ 0.05). The BW and BCSs showed no significant differences between the CON and SC groups (*p* ≥ 0.05). Additionally, in the same group, there was no significant difference in the BW and BCS at each time point (*p* ≥ 0.05).

### 3.2. Hematology Indexes

The hematology indexes for each dog were examined on days 0, 21, and 42 (Table 3). The findings revealed that the interaction between and the main effects of *S. cerevisiae* supplementation and the trial time had no significant effect on the hematological indicators (*p* ≥ 0.05).

### 3.3. Coefficients of the Total Tract Apparent Digestibility of Nutrients

The nutrients’ apparent digestibility is shown in Table 4. The addition of *S. cerevisiae* to their diet did not influence the ATTD of DM, OM, EE, CP, or GE in the aged dogs (*p* ≥ 0.05).

### 3.4. Fecal Quality Score and Microbial Metabolite Concentration

Neither the diet treatment nor the trial time affected the fecal score, SCFA levels, or BCFA levels (*p* ≥ 0.05). Nevertheless, compared with the CON group, the ammonia concentration in the feces was reduced in the dogs consuming the SC diet (*p* < 0.05), see Table 5.

### 3.5. Antioxidant Indexes

**Figure 1:**
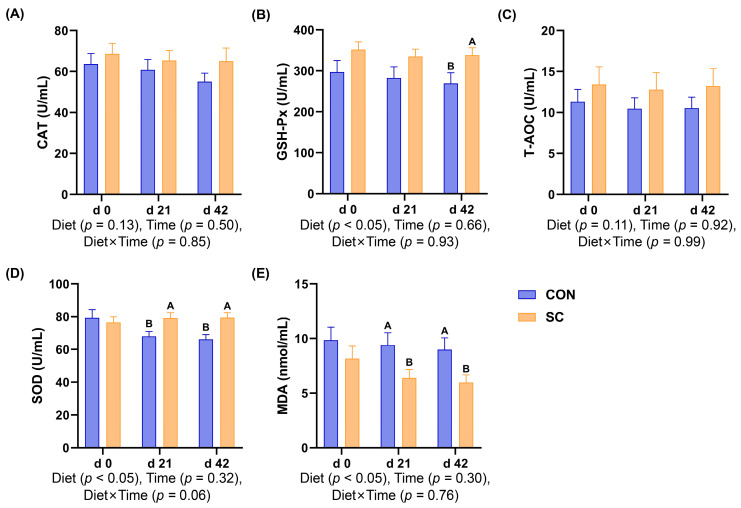
Bar charts showed the effects of diet treatment on the antioxidant indexes during the study period. (**A**) Catalase; (**B**) glutathione peroxidase; (**C**) total antioxidant capacity; (**D**) superoxide dismutase; (**E**) malondialdehyde. CON, a basal diet; SC, the basal diet with *Saccharomyces cerevisiae* (2 × 10^10^ CFU/kg of feed); *n* = 10. ^A,B^ Values with different superscripts refer to significant difference between the CON and SC groups at the time point (*p* < 0.05).

Figure 1 presents the serum antioxidant indexes of aged dogs subjected to different treatments or in different trial periods. The study period did not affect the antioxidant enzyme activities or MDA content (*p* ≥ 0.05). However, on day 42, the GSH-Px activity of the dogs fed the SC diet was higher than that of the dogs fed the CON diet (*p* < 0.05; Figure 1B). In addition, on d 21 and 42, compared with the dogs consuming the SC diet, the MDA content was enhanced while the SOD activity was inhibited in the dogs consuming the CON diet (*p* < 0.05; Figure 1D,E).

### 3.6. Inflammatory Factors

**Figure 2:**
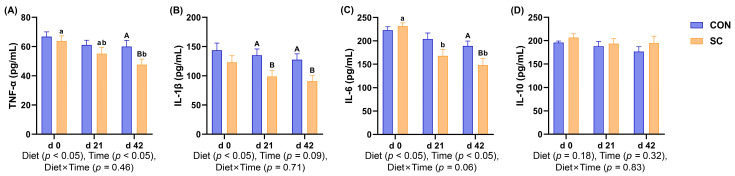
Bar charts showed the effects of diet treatment on the inflammatory factors during the study period. (**A**) Tumor necrosis factor-α; (**B**) interleukin-1β; (**C**) interleukin-6; (**D**) interleukin-10. CON, a basal diet; SC, the basal diet with *Saccharomyces cerevisiae* (2 × 10^10^ CFU/kg of feed); *n* = 10. ^A,B^ Values with different superscripts refer to significant difference between the CON and SC groups at the time point (*p* < 0.05). ^a,b^ Values with different superscripts mean difference between days in a group (*p* < 0.05).

In Figure 2, compared with the dogs fed the CON diet, the dogs fed the SC diet exhibited decreased TNF-α and IL-6 levels in their serum on d 42 (*p* < 0.05). Additionally, the IL-1β levels were lower in the SC treatment than those in the CON treatment on d 21 and 42 (*p* < 0.05). Moreover, the TNF-α levels were lower in the SC group on d 42 than those on d 0 (*p* < 0.05), and the levels of IL-6 were decreased on d 21 and 42 compared with d 0 (*p* < 0.05). However, the IL-10 levels had no change with different study periods or diet treatments (*p* ≥ 0.05).

### 3.7. Intestinal Barrier Function Parameters

**Figure 3:**
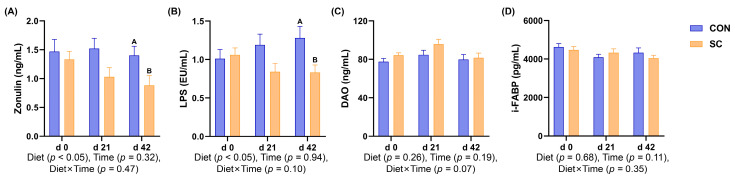
Bar charts showed the effects of diet treatment on the intestinal barrier function parameters levels during the study period. (**A**) Zonulin; (**B**) lipopolysaccharide; (**C**) diamine oxidase; (**D**) intestinal fatty acid-binding protein. CON, a basal diet; SC, the basal diet with *Saccharomyces cerevisiae* (2 × 10^10^ CFU/kg of feed); *n* = 10. ^A,B^ Values with different superscripts refer to significant difference between the CON and SC groups at the time point (*p* < 0.05).

The intestinal barrier function indicators of the aged dogs in the CON and SC groups during the study period are presented in Figure 3. On d 42, the levels of zonulin and LPS were lower in the dogs fed the SC diet than those in the dogs fed the CON diet (*p* < 0.05).

### 3.8. Fecal Microbiota Composition and Structure

**Figure 4:**
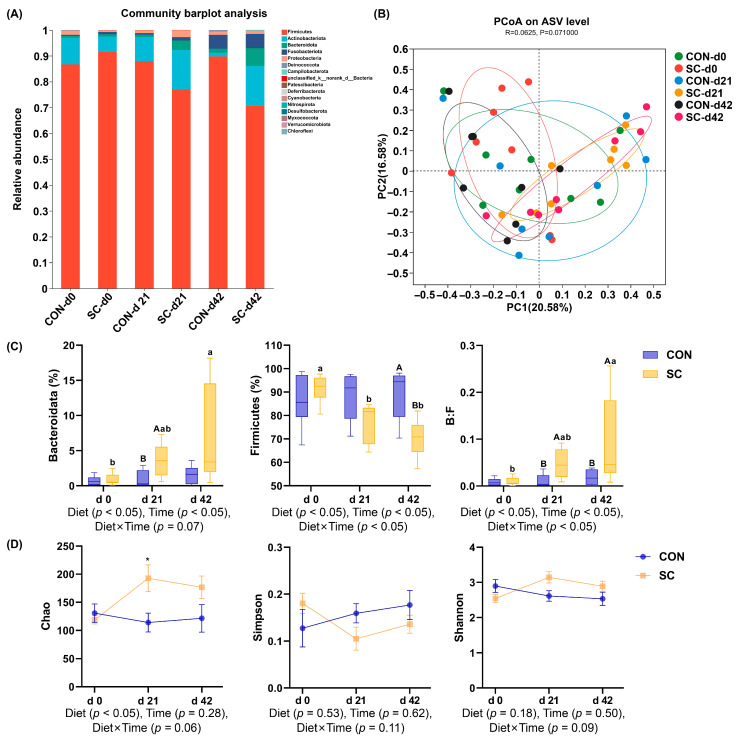
Alterations in the fecal microbiota structure of aged dogs subjected to different treatments during the study period. (**A**) The relative abundance of fecal microflora at the phylum level; (**B**) principal component analysis at the ASV level; (**C**) the relative abundance of Bacteroidetes and Firmicutes and the value of Bacteroidetes:Firmicutes; (**D**) the α-diversity analysis utilizing the Chao, Simpson, and Shannon indexes. CON, a basal diet; SC, the basal diet with *Saccharomyces cerevisiae* (2 × 10^10^ CFU/kg of feed); *n* = 10. ^A,B^ Values with different superscripts refer to significant difference between the CON and SC groups at the time point (*p* < 0.05). ^a,b^ Values with different superscripts refer to difference between days in a group (*p* < 0.05). * Superscripts refer to significant difference between the CON and SC groups at the time point (*p* < 0.05).

The bacterial composition at the phylum level of the aged dogs in different dietary treatments during the study period is presented in Figure 4A. The PCoA indicated that at the ASV level, no significant separation was shown in the clustering of fecal microbial communities among the two treatments (*p* ≥ 0.05; Figure 4B). On d 21, at the phylum level (Figure 4C), the fecal bacteria of the dogs fed the SC diet exhibited a higher abundance of Bacteroidetes than the dogs fed the CON diet, while the abundance of Firmicutes was lower in the SC treatment than that in the CON treatment on d 42. Meanwhile, in the SC group, the abundance of Bacteroidetes at d 42 was higher than that at d 0 (*p* < 0.05), and the abundance of Firmicutes showed a significant decrease by d 21 and 42 compared to that at d 0 (*p* < 0.05). Moreover, the ratio of Bacteroidetes to Firmicutes in the SC treatment significantly increased compared to that in the CON treatment on d 21 and 42 (*p* < 0.05), and the ratio of Bacteroidetes to Firmicutes in the dogs fed the SC diet increased significantly over time (*p* < 0.05; Figure 4C). Compared with the dogs fed the CON diet, the Chao index showed a significant increase in the dogs fed the SC diet by d 21 (*p* < 0.05, Figure 4D). Neither diet treatment nor trial period affected the Shannon or Simpson indexes (*p* ≥ 0.05; Figure 4D).

### 3.9. The Relative Abundance of Fecal Microbiota at the Family Level

**Figure 5:**
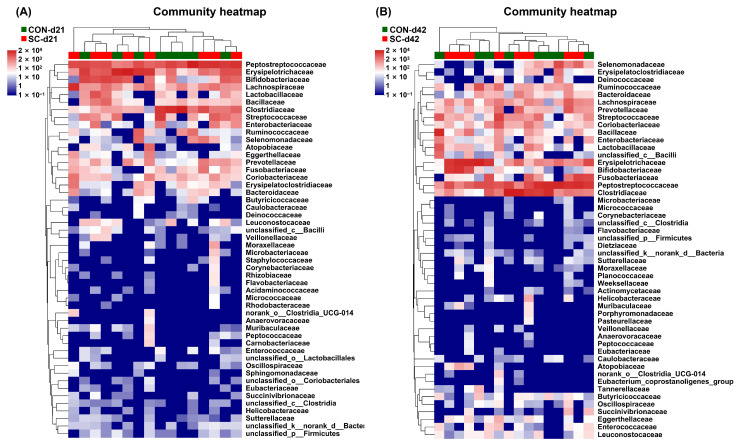
The relative abundance of fecal microbiota at the family level on d 21 and 42. (**A**) The relative abundance of fecal microbiota at the family level on d 21; (**B**) the relative abundance of fecal microbiota at the family level on d 42. CON, a basal diet; SC, the basal diet with *Saccharomyces cerevisiae* (2 × 10^10^ CFU/kg of feed); *n* = 10.

Figure 5 presents changes in the fecal microbiota composition at the family level in the aged dogs after different treatments. On d 21, the abundance of Prevotellaceae and Eggerthellaceae decreased in the dogs fed the CON diet compared with that in the dogs fed the SC diet (*p* < 0.05; Figure 5A). Moreover, on d 42, the abundance of Lachnospiraceae and Prevotellaceae in the CON group decreased compared to that in the SC group (*p* < 0.05; Figure 5B).

### 3.10. The Relative Abundance of Fecal Microbiota at the Genus Level

**Figure 6:**
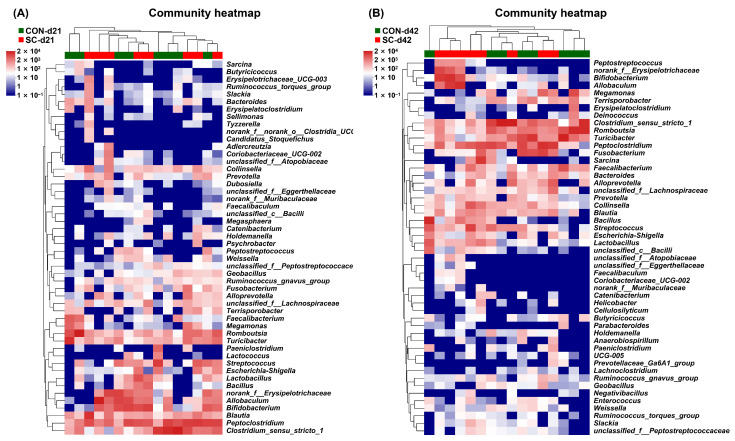
The relative abundance of fecal microbiota at the genus level on d 21 and 42. (**A**) The relative abundance of fecal microbiota at the genus level on d 21; (**B**) the relative abundance of fecal microbiota at the genus level on d 42. CON, a basal diet; SC, the basal diet with *Saccharomyces cerevisiae* (2 × 10^10^ CFU/kg of feed); *n* = 10.

Figure 6 illustrates alterations in the fecal microbiota composition at the genus level of the aged dogs following different treatments. The abundance of *Alloprevotella* was lower in the CON group than in the SC group on d 21 (*p* < 0.05; Figure 6A). Additionally, compared with that in the dogs fed the SC diet, the abundance of *Romboutsia* and *Terrisporobacter* was increased in the dogs fed the CON diet by d 42 (*p* < 0.05; Figure 6B).

## 4. Discussion

The gut microbiota can help protect against intestinal pathogens, improve barrier function, and strengthen immune function, all of which are crucial for supporting intestinal homeostasis [25]. Imbalance or dysfunction of the gut microbiota is linked to intestinal diseases in aged dogs, characterized by decreased microbial diversity and abundance, along with alterations in the microbiome composition and metabolite profiles [4,5]. Probiotics offer a potential pathway to manipulate the intestinal flora. Probiotics based on *S. cerevisiae* have been shown to be functional dietary supplements that have positive effects on the gut in humans and animals by improving the host’s intestinal immune response and regulating the gut microbiota [19,20]. However, previous studies have not investigated the influence of oral *S. cerevisiae* on the antioxidant capacity, inflammation status, intestinal barrier integrity, or microbiome composition in aged dogs.

The stability of reactive oxygen species (ROS) is essential for supporting regular physiological functions and cell redox homeostasis by regulating the production and scavenging of free radicals, and ROS can also create a suitable microenvironment for the proper activities of biological macromolecules [26]. The scavenging of free radicals depends on antioxidant capacity. When there is a disproportion between the generation of free radicals and the antioxidant network, this can result in apoptosis, tissue injury, and the emergence of various diseases [27,28]. GSH-Px and SOD are typical antioxidant enzymes and play an important role in preventing the damage caused by oxidative stress, while MDA is generated as a metabolite of lipid peroxidation in response to oxidative stress and directly reflects the level of lipid peroxidation [29,30]. Changes in the gut microbiota have been shown to alter age-related oxidative stress states [31]. As for *S. cerevisiae*, European seabass fed live *S. cerevisiae* had higher levels of SOD and GSH-Px in addition to lower MDA levels than fish on a basic diet, which were accompanied by downregulated heat shock protein 70 gene expression and markedly improved hematobiochemical and immune performance [32]. In addition, the inclusion of dietary *S. cerevisiae* increased the total antioxidant capacity and suppressed a rise in MDA and protein carbonyl derivatives caused by oxidative damage under cadmium stress in crayfish [33]. Furthermore, another study assessed the impact of a combination of live *S. cerevisiae* with yeast postbiotics and selenium-fortified yeast on ewes. The results indicated that dietary supplementation with probiotic and postbiotic yeast supplements enhanced their milk constituents and energy levels, improved their oxidative status, suppressed pro-inflammatory gene levels, and increased SOD and T-AOC levels during the peripartum period [34]. In this study, we found that adding *S. cerevisiae* to their diet significantly reduced the dogs’ MDA levels and increased their levels of GSH-Px and SOD. These results suggest that incorporating live *S. cerevisiae* could improve the antioxidant capacity of aged dogs.

The intestinal tract is the largest immune organ and regulates the occurrence and development of inflammation by maintaining host immunity homeostasis and tolerance, thus preventing pathological immune responses. The cells of the immune system produce cytokines, which mediate a variety of immune responses through their biological effects [35]. The equilibrium between pro-inflammatory cytokines and anti-inflammatory cytokines is crucial to sustaining normal immune status and physiological activities. IL-6, TNF-α, and IL-1β are typical pro-inflammatory cytokines that promote inflammatory reactions and stimulate immune active cells [36]. Another study has shown that *S. cerevisiae* significantly decreased the TNF-α concentrations and exhibited a tendency to reduce the IL-1β levels in the serum of dairy cows [37]. TNF-α and IL-1β have been demonstrated to exhibit pleiotropic effects, and their local activation can lead to an increase in the levels of the secondary inflammatory mediator IL-6. Research has demonstrated that using dietary live yeast cultures as a feed additive can improve the gut integrity and upper intestinal morphology, as well as reduce serum levels of IL-1β, TNF-α, and IL-6, in broiler chickens [38]. In addition, vacuoles obtained from *S. cerevisiae* could serve as a promising treatment option for modulating inflammation, and it was revealed that these vacuoles inhibited the nuclear factor kappa-B signaling pathway and decreased the levels of IL-1β, TNF-α, and IL-6 in LPS-stimulated macrophages [39]. Importantly, the immune effects of *S. cerevisiae* may be partially attributed to MOS and β-glucans, which are fundamental components of yeast’s cell walls. In yeast, MOS and β-glucan are functional polysaccharide components, each constituting approximately 30% of the dry weight of the cell wall, maintaining the morphology of yeast cells [40]. MOS has been shown to possess antigenic properties and can induce an immune response in animals. Adding MOS to the diet favorably promoted T cell and innate immune capabilities in piglets affected by porcine reproductive and respiratory syndrome virus, accompanied by improved growth performance and reduced concentrations of TNF-α [41]. In addition, MOS has been shown to prevent intestinal inflammation by directly interacting with the MOS receptors present in macrophages [42]. Yeast β-glucan can enhance humoral immunity and activate key immune cells involved in nonspecific and specific immunity, including helper B cells and T cells. Stimulated helper T cells secrete cytokines that support the B cell response and trigger the body to initiate an immunological reaction [43]. In terms of anti-inflammation, yeast β-glucan can reduce the levels of TNF-α, IL-6, and IL-1 to modulate inflammation [44,45]. In this study, we found that the addition of dietary *S. cerevisiae* significantly reduced the release of pro-inflammatory factors, including TNF-α, IL-1β, and IL-6, compared with that in the CON group. These results indicate that *S. cerevisiae* was capable of enhancing the immune state of the aged dogs.

The integrity of the gut epithelial layer serves as the primary physical barrier against the invasion of pathogenic antigens and is crucial for intestinal homeostasis and normal gut function [46]. Dietary supplementation with *S. cerevisiae* for 21 and 42 days was found to improve the intestinal barrier function of the ileum in Peking ducks, evidenced by increased mRNA expression levels of claudin-3, i-FABP, occludin, and ZO-1 [47]. Another study demonstrated that *S. cerevisiae* increased the relative content of gut barrier proteins, such as ZO-1, occludin, and claudin-1, and improved the integrity of the gut morphology in mice with ulcerative colitis [48]. In addition, *S. cerevisiae* enhanced the quantity of goblet cells and the expression of intestinal tight junction proteins in mice under induced *Fusobacterium nucleatum* infection and sodium glucan sulfate treatment, thereby further alleviating colonic inflammation [49]. In this study, we used canine serum indicators, including LPS, i-FABP, DAO, and zonulin, to measure intestinal barrier function. LPS is a distinctive constituent found in the cell wall of Gram-negative bacteria and can stimulate the host immune cells to produce inflammatory cytokines [50]. When the intestinal barrier is compromised, LPS in the intestinal lumen can enter the peripheral circulation and trigger or exacerbate inflammation in the body [51]. DAO, a highly reactive intracellular enzyme, and i-FABP, a soluble protein, are both located in the upper small intestinal mucosal villi in mammals. Zonulin is the sole known physiological regulatory protein for intestinal permeability [52,53,54]. When the intestinal permeability increases due to the stimulation of bacteria and antigens, DAO, i-FABP, and zonulin are secreted into the enteric cavity and subsequently into the bloodstream. Therefore, the concentrations of DAO, zonulin, and i-FABP in serum can reflect the integrity of and the degree of damage to the intestinal mechanical barrier [52,53,54]. In this study, dietary *S. cerevisiae* significantly decreased the serum levels of LPS and zonulin in the aged dogs, suggesting that *S. cerevisiae* could improve the intestinal barrier function and potentially maintain gut health in aged dogs.

Studies have demonstrated the influence of the intestinal microbiota and their metabolites on pet health and disease conditions [55,56]. The phyla of canine intestinal flora mainly include Firmicutes, Bacteroidetes, Proteobacteria, Actinobacteria, and Fusobacteria [3,57], which is consistent with our research. However, alterations in the intestinal microbiota due to aging further aggravate a decrease in immune function and an increase in intestinal permeability [6]. In our study, the ratio of Bacteroidetes to Firmicutes of the dogs fed the SC diet significantly increased compared with that of the dogs fed the CON diet by days 21 and 42, and the ratio of Bacteroidetes to Firmicutes increased over time in the SC group. Compared with young mice, the ratio of Firmicutes and Bacteroidota in the gut of aged mice over 1 year old was significantly reduced, accompanied by lipid and glucose metabolic disorders and liver damage [58]. Another study showed that mice receiving microbiota from aged or young mice exhibited significant changes in their gut microbial diversity and composition. At the phylum level, mice that received microbiota from aged mice had an increased ratio of Firmicutes to Bacteroidota [59]. Moreover, we found that the abundance of Lachnospiraceae and Prevotellaceae of the dogs fed the SC diet showed a significant increase compared with the dogs fed the CON diet on day 42. The Lachnospiraceae family constitutes the central component of the intestinal microbiota, colonizing the intestinal tract from birth and rising throughout the life of the host [60]. Lachnospiraceae exhibit strong hydrolytic activity and can utilize polysaccharides from dietary sources, including arabinoxylan, inulin, and starch, to produce SCFAs [61]. Prevotellaceae can degrade proteins and carbohydrates from the diet, and it has been reported that the relative abundance of members of Prevotellaceae exhibits a positive relationship with anti-inflammatory functions [62,63]. Therefore, these microbial analysis results suggest that the supplementation of *S. cerevisiae* in the diet is beneficial for the gut microbiota composition in aged dogs.

In fact, processes associated with aging affect the variability in the intestinal microbiota, potentially resulting in immune function impairment and metabolic alterations [64]. The composition of the intestinal microbiota changes in aged human and animals, generally indicating a decline in microbial diversity and abundance [65,66]. The intake of certain probiotics has been shown to enhance microbial richness and diversity in aged animals, thereby improving the intestinal environment and regulating immune function [67]. In addition, 12-month supplementation with *Lactobacillus reuteri* ATCC PTA 6475 significantly increased the richness of the gut microbiota at the genus level and inhibited the concentrations of inflammatory factors in elderly females with a decreased bone mineral density [68]. Similarly, orally administering *Lactobacillus* or *Bifidobacterium* to aged mice for 12 weeks enhanced the variety of intestinal microbiota and altered the microbiota composition, which was associated with reduced inflammation in the peripheral tissues and improved hepatic lipid accumulation [69]. In our study, the Chao index of the aged dogs fed the SC diet significantly increased compared with that of the aged dogs fed the CON diet by day 21, suggesting dietary *S. cerevisiae* could increase gut microbial community richness. However, the diversity of the gut microbiota in aged dogs remained unchanged following the intake of *S. cerevisiae*, which could be attributed to the initial gut health status of the dogs and the trial duration.

## 5. Conclusions

In conclusion, dietary *S. cerevisiae* could decrease pro-inflammatory factor levels, improve antioxidant capacities, and enhance gut barrier function in dogs in an aging state. Furthermore, dietary *S. cerevisiae* increased the gut microbial richness and altered the microbiome composition of aged dogs, reflected in the inhibited abundance of Firmicutes and the enriched abundance of Bacteroidetes, Lachnospiraceae, and Prevotellaceae. These results provide support for the application of dietary *S. cerevisiae* in companion animals to improve their gut barrier function and gut health in old age.

## Figures and Tables

**Table 1:** Nutrient composition and concentrations of the basal diets (expressed as a percentage on an as-fed basis).

Items	Content
Ingredient, %	
Chicken meal	30.00
Pea	7.00
Peeled barley	13.50
Chicken liver meal	5.00
Tapioca starch	15.00
Chicken oil	8.50
Sweet potato granules	8.00
Fish meal	6.00
Carrot powder	1.00
Pumpkin powder	2.00
Fish oil	2.50
Premix ^1^	1.50
Total	100.00
Nutrient levels	
Metabolic energy, kcal/kg	4.235
Crude protein, %	32.85
Ether extract, %	18.32
Calcium, %	1.23
Phosphorus, %	0.97

Note: ^1^ The premix per kg supplied the following content in the diet: vitamin A = 15,000 IU, vitamin B_1_ = 30 mg, vitamin B_2_ = 28 mg, vitamin B_3_ = 110 mg, vitamin B_5_ = 85 mg, vitamin B_6_ = 12 mg, vitamin B_12_ = 0.19 mg, vitamin D_3_ = 15 IU, vitamin E = 75,300 IU, Co (CoSO_4_) = 0.10 mg, Cu (CuSO_4_) = 3 mg, Fe (FeSO_4_) = 50 mg, I (CaI_2_) = 40 mg, Mn (MnSO_4_) = 18 mg, Na (Na_2_SeO_3_) = 0.05 mg, Zn (ZnSO_4_) = 38 mg, Se (Na_2_SeO_3_) = 260 mg.

**Table 2:** Body performance of aged dogs in different diet groups during the study period *.

Items	CON			SC			SEM	*p*-Value		
	Day 0	Day 21	Day 42	Day 0	Day 21	Day 42		Diet	Time	Diet × Time
Body weight, kg	28.39	28.44	28.59	29.09	29.42	29.73	0.50	0.37	0.95	0.99
Daily intake, g/d	417.25	427.67	420.03	418.00	406.56	426.34	4.86	0.64	0.86	0.50
Body condition score	4.80	4.90	5.10	4.80	4.80	4.90	0.09	0.56	0.63	0.90

* CON, a basal diet; SC, the basal diet with *Saccharomyces cerevisiae* (2 × 10^10^ CFU/kg of feed); *n* = 10.

**Table 3:** Effects of dietary *Saccharomyces cerevisiae* on hematology indexes in aged dogs during the study period *.

Items	CON			SC			SEM	*p*-Value		Reference Interval
	Day 0	Day 21	Day 42	Day 0	Day 21	Day 42		Diet	Time	
Hematological indexes										
WBCs, 10^9^/L	13.83	14.94	14.48	15.23	12.51	10.76	0.52	0.16	0.24	6.00~16.9
Lymphocytes, 10^9^/L	3.58	3.40	3.67	3.76	3.58	2.82	0.22	0.81	0.65	1.10~6.30
Lymphocytes, %	18.24	19.01	18.88	18.24	18.44	15.91	0.47	0.17	0.49	12.0~30.0
Monocytes, 10^9^/L	0.92	0.89	0.94	0.92	0.86	0.86	0.05	0.63	0.84	0.15~1.35
Gran, 10^9^/L	9.09	9.05	8.81	10.39	9.43	9.52	0.40	0.55	0.62	6.20~14.8
Monocytes, %	6.77	5.97	7.35	6.38	5.29	5.57	0.29	0.07	0.35	3.00~10.0
Gran, %	70.81	73.98	73.51	70.55	69.75	69.16	0.97	0.12	0.9	63.0~87.0
RBCs, 10^12^/L	6.48	6.08	6.33	6.14	6.25	5.76	0.17	0.67	0.65	5.50~8.50
HGB, g/L	151.16	165.49	157.47	139.30	137.90	149.14	5.33	0.2	0.73	120~180
PLTs, 10^9^/L	294.10	291.10	303.38	328.15	324.82	306.31	13.15	0.85	0.97	175~500
RDW, %	12.83	12.57	12.85	13.61	13.04	13.00	0.19	0.09	0.57	12.0~21.0
MCHC, g/L	344.08	333.66	343.93	354.92	350.34	351.23	3.56	0.16	0.57	300~369
MPV, fL	9.46	9.23	9.55	9.59	9.34	8.89	0.16	0.67	0.62	6.70~11.1
Biochemical indexes										
ALT, U/L	38.05	36.01	40.73	40.80	38.64	43.68	1.94	0.27	0.51	10.0~100
AST, U/L	26.65	25.18	28.49	34.46	32.63	36.88	2.06	0.09	0.58	0~50.0
GLU, mmol/L	5.30	5.00	5.97	6.26	5.61	5.81	0.15	0.05	0.12	3.89~7.94
BUN, mmol/L	3.44	3.13	3.56	3.66	3.35	3.80	0.12	0.31	0.32	3.20~7.00
ALP, U/L	152.40	144.66	163.49	179.26	170.18	186.32	7.52	0.05	0.49	23.0~212
Cr, μmol/L	63.78	61.41	57.39	51.24	48.55	54.89	2.76	0.08	0.9	44.0~159
TCHO, mmol/L	4.31	3.96	4.51	4.69	4.33	4.93	0.17	0.31	0.31	2.84~8.27
TP, g/L	65.62	61.41	69.40	73.82	67.01	74.11	1.95	0.13	0.22	52.0~82.0
ALB, g/L	32.26	30.53	34.53	31.57	29.85	33.76	0.87	0.72	0.18	22.0~39.0

* CON, a basal diet; SC, the basal diet with *Saccharomyces cerevisiae* (2 × 10^10^ CFU/kg of feed); *n* = 10; WBC, white blood cell; RBC, red blood cell; HGB, hemoglobin; PLT, platelet; RDW, red blood cell distribution width; MCHC, mean corpuscular hemoglobin concentration; MPV, mean platelet volume; ALT, alanine transaminase; AST, aspartate amino transferase; GLU, glucose; BUN, blood urea nitrogen; ALP, alkaline phosphatase; Cr, creatinine; TCHO, total cholesterol; TP, total protein; ALB, albumin.

**Table 4:** Effects of dietary *Saccharomyces cerevisiae* on coefficients of total tract apparent digestibility of nutrients in aged dogs during the study period *.

Digestibility (%)	CON	SC	SEM	*p*-Value
Dry matter	84.53	83.87	0.66	0.52
Organic matter	85.61	86.97	0.75	0.21
Crude protein	86.90	85.14	0.86	0.15
Ether extract	90.69	90.52	0.85	0.88
Gross energy	85.34	87.13	1.04	0.22

* CON, a basal diet; SC, the basal diet with *Saccharomyces cerevisiae* (2 × 10^10^ CFU/kg of feed); *n* = 10.

**Table 5:** Fecal characteristics and microbial metabolite concentration in aged dogs during the study period *.

Items	CON			SC			SEM	*p*-Value		
	Day 0	Day 21	Day 42	Day 0	Day 21	Day 42		Diet	Time	Diet × Time
Fecal score	2.90	2.60	2.70	2.70	2.50	2.70	0.11	0.67	0.68	0.94
Short-chain fatty acids (µmol/g dry matter basis)										
Acetate	351.16	336.52	332.97	335.39	348.55	344.87	6.20	0.83	0.96	0.60
Propionate	149.01	144.68	151.41	157.06	155.03	158.43	4.69	0.39	0.91	0.99
Butyrate	82.53	79.23	80.98	80.26	89.93	83.63	1.39	0.19	0.63	0.16
Valerate	3.48	3.65	4.01	3.08	3.42	3.74	0.20	0.47	0.51	0.99
Branched-chain fatty acids (µmol/g dry matter basis)										
Isovalerate	9.98	9.51	9.77	9.34	9.85	9.81	0.16	0.80	0.94	0.47
Isobutyrate	7.25	6.47	7.79	7.38	7.12	6.50	0.23	0.71	0.65	0.22
Ammonia, μmol/g	179.04	173.67	169.53 ^A^	172.89	152.83	132.46 ^B^	4.95	<0.05	0.10	0.41

* CON, a basal diet; SC, the basal diet with *Saccharomyces cerevisiae* (2 × 10^10^ CFU/kg of feed); *n* = 10. In the same row, values marked with ^A,B^ signify a significant difference between the CON and SC groups at the time point (*p* < 0.05).

## Data Availability

Data are contained within the article.
